# Therapy of IgA nephropathy: time for a paradigm change

**DOI:** 10.3389/fmed.2024.1461879

**Published:** 2024-08-15

**Authors:** Jonathan Barratt, Richard A. Lafayette, Jürgen Floege

**Affiliations:** ^1^Department of Cardiovascular Sciences, University of Leicester and Leicester General Hospital, Leicester, United Kingdom; ^2^Division of Nephrology, Stanford University Medical Center, Stanford, CA, United States; ^3^Division of Nephrology and Rheumatology, Department of Cardiology, RWTH Aachen University Hospital, Aachen, Germany

**Keywords:** IgA nephropathy, proteinuria, nephron, eGFR, sparsentan, SGLT2 inhibitor, glucocorticoids, budesonide

## Abstract

Immunoglobulin A nephropathy (IgAN) often has a poor outcome, with many patients reaching kidney failure within their lifetime. Therefore, the primary goal for the treatment of IgAN should be to reduce nephron loss from the moment of diagnosis. To achieve this, IgAN must be recognized and treated as both a chronic kidney disease and an immunological disease. Agents that have received US Food and Drug Administration and European Medicines Agency approval for the treatment of IgAN include modified-release/targeted-release formulation budesonide (Nefecon) and sparsentan, a selective dual endothelin-A and angiotensin II receptor type 1 antagonist. Other agents, including selective endothelin receptor antagonists, selective or combined APRIL and BAFF antagonists, and a vast array of complement inhibitors are being investigated for the treatment of IgAN. Furthermore, treatment combinations are also being studied, including sodium–glucose cotransporter-2 inhibitors with endothelin receptor antagonists. Due to the complexity of IgAN, combination treatment, rather than a single-agent approach, may provide maximum benefit. With the number of treatments for IgAN likely to increase, combinations allowing safe and effective treatment to halt progression to kidney failure seem within grasp. While trials evaluating combinations are ongoing, more are needed to pave the way for a comprehensive IgAN treatment strategy. Furthermore, an approach to IgAN treatment in which agents are combined early to achieve rapid induction of remission and prevent unnecessary and irreversible nephron loss is required. Following remission, treatments may be adjusted and stripped back as necessary in the maintenance phase with close monitoring. This review discusses the current status of IgAN treatment and explores future strategies to improve outcomes for patients with IgAN.

## Introduction

Immunoglobulin A nephropathy (IgAN) is the most common primary glomerulonephritis worldwide, with an incidence rate of at least 2.5/100,000 adults per year (1–3). Due to the progressive nature of IgAN, patients generally face a poor prognosis if the condition is not appropriately controlled. While IgAN can be a slowly progressive disease (3), approximately 30–40% of patients will develop kidney failure within 10 years of diagnosis, increasing to more than 50% within 20 years (4, 5). Data from the UK National Registry of Rare Kidney Diseases (RaDaR) highlighted that even patients traditionally regarded as being at low risk, with urine protein-to-creatinine ration (UPCR) <0.88 g/g (<100 mg/mmol), had high rates of kidney failure within 10 years of diagnosis. Furthermore, this analysis found that almost all patients were likely to experience kidney failure within their expected lifetime, and that patients with modest rates of estimated glomerular filtration rate (eGFR) decline have a high risk of progression to kidney failure (4). Treatment to reduce eGFR loss to <1 ml/min per 1.73 m^2^ per year from diagnosis may change this trajectory (4). A paradigm shift in the diagnosis and treatment of IgAN is therefore required.

One core challenge is that IgAN is often asymptomatic, and therefore the majority of patients are diagnosed by chance and often late in the disease course, with significant proteinuria and an impaired GFR at presentation (3, 5). At diagnosis, at least 50% of patients will have stage 3 or greater chronic kidney disease (CKD), indicative of a significant loss of functioning nephrons (4–6). Earlier diagnosis is essential; however, biomarkers for early diagnosis are lacking (7). Urinalysis is not performed routinely in most countries and serum creatinine is not an optimal marker for identifying early kidney function decline (8, 9).

**Figure 1:**
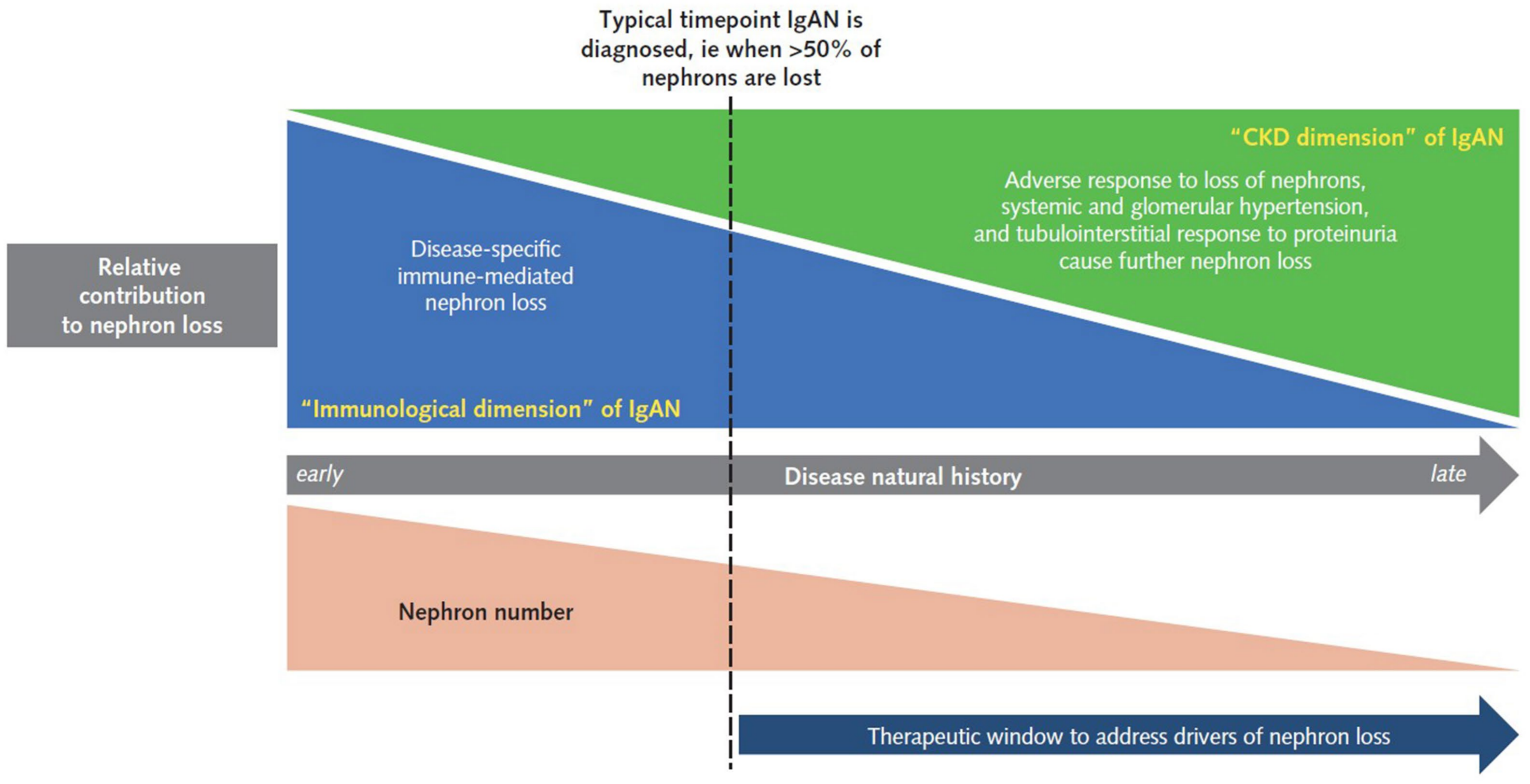
A stylized representation of the unmet need in a typical adult patient with IgAN. At the time a patient is typically diagnosed with IgAN, both disease-specific, immune-mediated nephron loss and universal CKD causes of nephron loss are at play. During the earlier stages of IgAN, often prior to diagnosis, the immunological aspects are the greatest contributor to disease progression. However, the adverse response to nephron loss through fundamental CKD responses, including systemic hypertension, glomerular hyperfiltration, and tubulointerstitial damage in response to proteinuria, which initially contribute little, become a greater factor as nephron loss increases. It is important to note that treatment approaches and aspirations will differ in some IgAN populations; for example, in pediatric patients, IgAN is often diagnosed earlier in the natural history, while in transplant patients there is the opportunity to detect recurrent disease early and therefore intervene before significant nephron loss has occurred. CKD, chronic kidney disease; IgAN, IgA nephropathy.

Until recently, treatment options were limited, and even now there is a high unmet need for therapies that are both effective in treating IgAN and well tolerated (10). To prevent further nephron loss and subsequent kidney function decline, IgAN treatment should address both the CKD and immunological dimensions of IgAN (Figure 1).

## Approaching IgAN treatment

### The CKD dimension of IgAN

There are several fundamental maladaptive responses that contribute to the decline in kidney function in IgAN. Key components include development of arterial and intraglomerular hypertension, the loss of filtration barrier integrity, increased proteinuria, and tubular interstitial fibrosis (11–13).

Nephron loss leads to homeostatic responses in the kidneys to maintain the GFR by increasing blood flow through the remaining nephrons, ie hyperfiltration. However, instead of maintaining glomerular function and structure, this process over time leads to further glomerular injury and ultimately amplifies glomerulosclerosis and loss of nephrons (14).

### The immunological dimension of IgAN

In addition to CKD processes, there are disease-specific processes involved in the development of IgAN. The pathogenesis of IgAN represented by the “Four Hit Hypothesis” has been described elsewhere (15–17). Briefly, the overproduction and consequent elevated circulating levels of galactose-deficient (Gd) IgA1 antibodies (Gd-IgA1; Hit 1), are associated with the formation of Gd-IgA1 complexes and/or the development of Gd-IgA1 autoantibodies that recognize the Gd-IgA1 hinge region O-glycans (Hit 2). Circulating Gd-IgA1-containing immune complexes are formed (Hit 3), some of which deposit in the mesangium (Hit 4) due to mesangial trapping/binding and the increased affinity of Gd-IgA1 for extracellular matrix components. Deposited Gd-IgA1 immune complexes bind to cell surface mesangial cell IgA receptors, triggering the release of inflammatory/fibrotic mediators, complement activation, and mesangial cell proliferation. These mediators promote monocyte/macrophage influx into the glomerulus, mesangial matrix production, and activation and injury to podocytes and tubular epithelial cells (15–17).

## Therapies addressing the CKD dimension

### Current options included in the KDIGO 2021 clinical practice guideline for IgAN

Current Kidney Disease Improving Global Outcomes (KDIGO) guidelines recommend optimized supportive care for patients with IgAN to preserve kidney function and reduce cardiovascular (CV) risk, including optimal control of blood pressure (BP), use of the maximum tolerated dose of renin–angiotensin system inhibitors (RASi), restriction of dietary sodium intake, normalization of body weight and smoking cessation (3). Despite these recommendations, RASi are often used suboptimally. For example, in the recently completed Phase 3 PROTECT study, ~60% of patients were reported to be receiving maximally labeled or tolerated doses of an angiotensin-converting enzyme inhibitor/angiotensin receptor blockers prior to randomization (18). However, in the irbesartan arm of the study, 97% of patients were then titrated to the maximum recommended dose (18). Interestingly, eGFR decline in the irbesartan arm was slower compared with most other randomized controlled trials in IgAN (18).

### Emerging and recently approved treatments

Beyond RASi, novel agents to be considered as treatment for the CKD dimension of IgAN include sodium–glucose cotransporter-2 inhibitors (SGLT2is), dual endothelin angiotensin receptor antagonists (DEARAs), endothelin receptor antagonists (ERAs) and mineralocorticoid receptor antagonists (MRAs). Some of these agents have multiple mechanisms of action and could be considered to act upon both the CKD and immunological dimensions of IgAN (18–20).

#### SGLT2i

Results from trials of SGLT2i in non-diabetic CKD indicate a role for this class of drug in the treatment of IgAN (21, 22). In a prespecified analysis of patients with IgAN included in the DAPA-CKD trial, dapagliflozin significantly lowered the risk of the primary composite outcome (sustained decline in eGFR of ≥50%, end-stage kidney disease [ESKD], or death from a kidney disease-related or CV cause) (21). The EMPA-KIDNEY study, which included a broad range of CKD patients, including >800 with IgAN (23), showed that empagliflozin reduced the risk of the composite outcome of kidney disease progression or CV death compared with placebo (22). Together, DAPA-CKD and EMPA-KIDNEY support the broad use of SGLT2i in the treatment of CKD, including in IgAN patients who have reduced kidney function and persistent proteinuria.

#### DEARAs and ERAs

In the Phase 3, active-controlled PROTECT trial assessing the DEARA sparsentan (a selective dual endothelin-A receptor [ET_A_R] and angiotensin II receptor type 1 [AT_1_R] antagonist) vs. the maximum labeled dose of irbesartan, sparsentan treatment significantly reduced proteinuria (by on average 40%) over the full 2 years of the study and slowed the rate of loss of kidney function compared with irbesartan (1.0–1.1 ml/min per 1.73 m^2^ per year difference between arms) (18). Notably, the benefits observed in PROTECT with sparsentan vs. irbesartan appear to be largely independent of BP, with only a modest BP difference reported (18). However, it is important to note that hypotension occurred in a greater proportion of patients receiving sparsentan than irbesartan (13% vs. 4%, respectively) (18). Sparsentan received US Food and Drug Administration accelerated approval for the treatment of IgAN in adult patients at risk of rapid disease progression in 2023 and European Commission conditional marketing authorization for the treatment of adults with primary IgAN with a urine protein excretion ≥1.0 g/day (or UPCR ≥0.75 g/g) ((24, 25)).

Clinical trials of ERAs in IgAN are beginning to report outcomes. Atrasentan, a selective ET_A_R antagonist, is being investigated in IgAN. The Phase 2 AFFINITY open-label basket trial included an IgAN cohort with patients required to be on maximally tolerated and stable doses of RASi. Interim data to Week 24 demonstrated a reduction in proteinuria with addition of atrasentan (26). The Phase 3 ALIGN trial of atrasentan in IgAN patients is ongoing (27).

#### MRA

The Phase 3 FIDELIO-DKD study in patients with CKD and type 2 diabetes showed that treatment with finerenone, a selective, non-steroidal MRA, lowered the risk of the primary outcome event (kidney failure, a sustained decrease of ≥40% in eGFR from baseline or death from renal causes) and also of the key secondary outcome event (death from CV causes, non-fatal myocardial infarction, non-fatal stroke or hospitalization for heart failure) compared with placebo (28). The ongoing FIND-CKD trial is investigating the effect of finerenone in patients with non-diabetic CKD (29) and may deliver evidence for yet another approach for IgAN treatment.

## Therapies addressing the immunological dimension

The key goals when addressing the immunological dimension of IgAN are (1) to stop the production of pathogenic IgA and the formation of circulating IgA immune complexes, (2) to switch off inflammatory pathways operating within the kidneys, and (3) to switch off pathways driving fibrosis within the kidneys. With the latter goal, we have made little progress in targeting profibrotic pathways in the kidneys and at present there is little on the horizon, particularly in IgAN.

### Current options included in the KDIGO 2021 clinical practice guideline for IgAN

For ~50 years following the description of IgAN in 1968 as a distinct disease, no progress was made in the development of therapies to stop the production of pathogenic IgA or inhibit pathways driving fibrosis within the kidneys (30). Efforts had focused on limiting glomerular inflammation with systemic glucocorticoids, as employed in other inflammatory glomerulonephritides; however, data from these initially small, low-quality clinical trials in IgAN were mixed (31).

In 2021, the KDIGO guideline suggested that a 6-month course of systemic glucocorticoids could be considered in patients with proteinuria >1 g/day despite optimized supportive care. However, only after consideration of enrollment in a clinical trial and discussion with the patient on the likely toxicity risks. It was also recommended that systemic glucocorticoids be avoided in patients with low eGFR, obesity, diabetes, chronic infection, and/or severe hypertension amongst other conditions (3).

Both the STOP-IgAN (32, 33) and TESTING (34) trials investigated systemic glucocorticoids for treating IgAN. In STOP-IgAN, Caucasian patients with a high risk of progression were randomized to receive supportive care alone or supportive care plus immunosuppressive therapy (32). After 3 years, a greater proportion of patients in the intervention group achieved complete clinical remission, which included a UPCR <0.2 vs. the supportive care only group (17 vs. 5%, respectively; *p* = 0.01). However, a decrease in eGFR of ≥15 ml/min per 1.73 m^2^ occurred in 26 vs. 28% receiving intervention vs. supportive care only, respectively. A greater number of patients receiving systemic glucocorticoids than supportive care alone suffered from severe infections, impaired glucose tolerance and weight gain ≥5 kg in the first year (32). Over a follow-up period of ≤10 years, analysis of an adapted primary endpoint did not find any difference in outcomes between the two arms (33).

In TESTING, almost exclusively in South-East Asian IgAN patients at high risk of progression, patients were treated with oral methylprednisolone or placebo for 6–9 months (34). Proteinuria was significantly lower in the methylprednisolone vs. placebo group during follow-up. Furthermore, treatment with methylprednisolone significantly reduced the composite outcome of kidney function decline, kidney failure, or death due to kidney disease compared with placebo (34). Despite reductions in the risk of the composite outcome, it is noteworthy that the reduction in proteinuria observed was no longer apparent ~2.5–3 years after randomization (34). Serious adverse events (AEs) reported in TESTING included several infection-related deaths, all occurring with methylprednisolone, and led to the trial being halted (34). TESTING was restarted after a pause with a lower dose of methylprednisolone plus antimicrobial prophylaxis (sulfamethoxazole-trimethoprim); this lower dose regimen resulted in a lower incidence of serious AEs while showing no significant heterogeneity in terms of treatment effect compared with the full-dose regimen (34).

Together, STOP-IgAN (32, 33) and TESTING (34) indicate that targeting glomerular inflammation with systemic glucocorticoids in the short term reduces proteinuria while on treatment; however, proteinuria increases upon treatment termination. Corticosteroid-related benefits for long-term kidney outcomes are controversial and AEs can be significant.

Immunosuppressive drugs, including cyclophosphamide, tacrolimus and azathioprine, targeting pathogenic IgA production, have been tested in IgAN patients, often in combination with low-dose glucocorticoids, but are not recommended in the guidelines due to unclear/insufficient evidence of efficacy and tolerability. There are some data to suggest mycophenolate mofetil (MMF) may be beneficial in Chinese patients, where it has been used as a glucocorticoid sparing agent (3, 35).

## Emerging and recently approved treatments

### Targeting pathogenic IgA synthesis

#### B-cell modulation

##### Modified-release/targeted-release formulation budesonide (Nefecon)

The first therapy widely approved for the treatment of IgAN is a modified-release/targeted-release formulation of the glucocorticoid budesonide, designed to locally suppress pathogenic IgA production in the gut-associated lymphoid tissue (GALT) of the terminal ileum (36, 37). Results from the Phase 3 NefIgArd trial found that a 9-month treatment course of Nefecon lowered UPCR and reduced eGFR loss compared with placebo (38). Over 2 years (9 months on treatment and 15 months observational follow-up), the time-weighted average of eGFR demonstrated a significant benefit with Nefecon vs. placebo (delta 5.05 ml/min per 1.73 m^2^ [95% CI, 3.24–7.38; *p* < 0.0001]), suggesting a clinically relevant reduction in eGFR decline (39). Furthermore, eGFR total slope over 2 years demonstrated a difference of 2.95 ml/min per 1.73 m^2^ per year (95% CI, 1.67–4.58; *p* < 0.0001) in favor of Nefecon (39). However, it should be noted that following the initial positive acute effect on eGFR in the first 3 months of treatment with Nefecon, eGFR decline in both arms continued at a more similar rate over the remaining period of the trial. Furthermore, 3 months after treatment termination, UPCR increased in the Nefecon arm, suggesting that continued suppression of the GALT is required to maintain suppression of pathogenic IgA production and deliver renoprotection, consistent with biomarker data reported from the NEFIGAN study (40).

##### Inhibition of BAFF/APRIL

A number of agents targeting the cytokines A proliferation-inducing ligand (APRIL) and B-cell activating factor (BAFF), which regulate B-cell maturation, function, and survival, are currently in Phase 2 or 3 trials in IgAN. Sibeprenlimab and zigakibart are monoclonal antibodies that block APRIL. In early-phase studies both have been shown to reduce levels of Gd-IgA1 and proteinuria, and slow loss of kidney function (41–44). Combination blockade of BAFF and APRIL is also being evaluated in studies of atacicept, telitacicept, and povetacicept, with early data showing reductions in serum Gd-IgA1 and proteinuria. There are also early data showing that atacicept and telitacicept slow kidney function loss (45–47).

##### B/plasma cell depletion

While CD20 (B-cell) depletion with rituximab was shown to be ineffective in IgAN (48), other depletion strategies are being evaluated in IgAN. Two studies are ongoing examining CD38 (plasma-cell) depletion with felzartamab (49) and mezagitamab (50). A small study of the proteasome inhibitor bortezomib, a plasma cell-depleting agent, suggested some benefit of plasma cell depletion in IgAN (51).

### Targeting inflammation

There is an increasing acceptance that systemic glucocorticoid use should be avoided or substantially limited in patients with kidney disease due to short-and long-term toxicity, and poor patient tolerability. There is emerging evidence that inhibition of complement activation may provide an equally efficacious, and more tolerable, alternative to systemic glucocorticoids in controlling inflammation in the kidneys. Inhibitors of the alternative, lectin, and final common pathways of complement are currently being evaluated in Phase 2 and Phase 3 clinical trials in IgAN (41, 42, 46, 52–54). Early indications are that inhibition of the alternative pathway with iptacopan appears to be beneficial in IgAN (53), while the Phase 3 ARTEMIS-IgAN trial of narsoplimab, a lectin pathway inhibitor, did not achieve the primary endpoint of reduction in proteinuria at 36 weeks vs. placebo (55).

## The future for clinical trials in IgAN

The key goal when managing IgAN patients is to stop or slow further nephron loss. As nephron loss is driven by different pathogenic pathways in IgAN, this will only be achieved with combination therapy; a single-agent approach will unlikely be sufficient to prevent disease progression (56). A number of different therapeutic strategies are being evaluated in IgAN with the hope that it will be possible to combine these into multitargeted therapeutic drug regimens capable of addressing the individual drivers of nephron loss in IgAN. Future IgAN studies will need to focus on the safety and effectiveness of combination regimens that include drugs with different, complementary mechanisms.

Pleasingly, studies are already underway to investigate new drug combinations in both CKD and IgAN. Numerous studies are evaluating RASi, SGLT2i, and ERA combination therapy. The PROTECT open-label extension sub-study will assess the safety and efficacy of dapagliflozin in combination with stable sparsentan treatment in IgAN patients (57). The Phase 2 SPARTACUS study is investigating sparsentan with stable SGLT2i (58). The Phase 2 ASSIST crossover study will evaluate the safety and efficacy of atrasentan vs. placebo in IgAN patients already on RASi and SGLT2i therapy (59). In the recently reported Phase 2b ZENITH-CKD trial, the ERA zibotentan, combined with dapagliflozin, reduced albuminuria compared with dapagliflozin plus placebo in patients with proteinuric CKD (60, 61), and a follow-on Phase 3 study is currently recruiting (62).

## A need to personalize IgAN treatment

IgAN is a highly heterogeneous disease, both within our own individual clinical practice, but also when considering the global pattern of disease (63–67). Therefore, a treatment approach tailored to a patient’s disease state and treatment needs is essential. The 2021 KDIGO guideline acknowledged this heterogeneity, and noted that there was some evidence for efficacy of MMF and hydroxychloroquine in Chinese IgAN patients which had not been replicated in Caucasians (3).

One of the major unanswered questions in IgAN remains why disease prevalence and severity increases as one moves from the West to the East, and why East and South Asians continue to experience poorer outcomes following migration to the Americas and Europe. It has been widely reported that the pattern of histopathological lesions in kidney biopsies and the response to therapeutic interventions can differ between ethnicities (63). Consistent with these observations, genome-wide association studies have demonstrated differences in the frequency of genome-wide significant risk loci for IgAN in different ethnicities, with a higher frequency of the identified risk loci in Chinese populations (63). As many of these risk loci involve pathogenic pathways currently being targeted by drugs in various phases of clinical development, it will be important in the future to consider ethnicity in treatment regimens.

A major area of unmet need in the management of IgAN is the availability of validated serum, urine and kidney biomarkers to direct treatment choice, to rigorously monitor treatment response and detect treatment-related toxicity (3, 56). There is a rapid growth in omics-based discovery studies in IgAN utilizing genomics, transcriptomics, and proteomics to identify biomarkers for IgAN diagnosis, prognostication, treatment selection and treatment monitoring (56, 68). At present, however, we do not have clinically validated serum or urine biomarkers (3), although numerous non-validated research assays are available to measure different factors, including Gd-IgA1, and assess the extent of intrarenal inflammation and activation of profibrotic pathways (56). If the goal is to truly personalize the approach to management, both for initiation and withdrawal of disease-modifying therapies, alone or in combination, we need easy-to-measure and accessible biomarkers that can be reliably introduced into routine clinical care. This must be a priority for researchers and the biotechnology industry over the coming years.

## Future concepts

**Figure 2:**
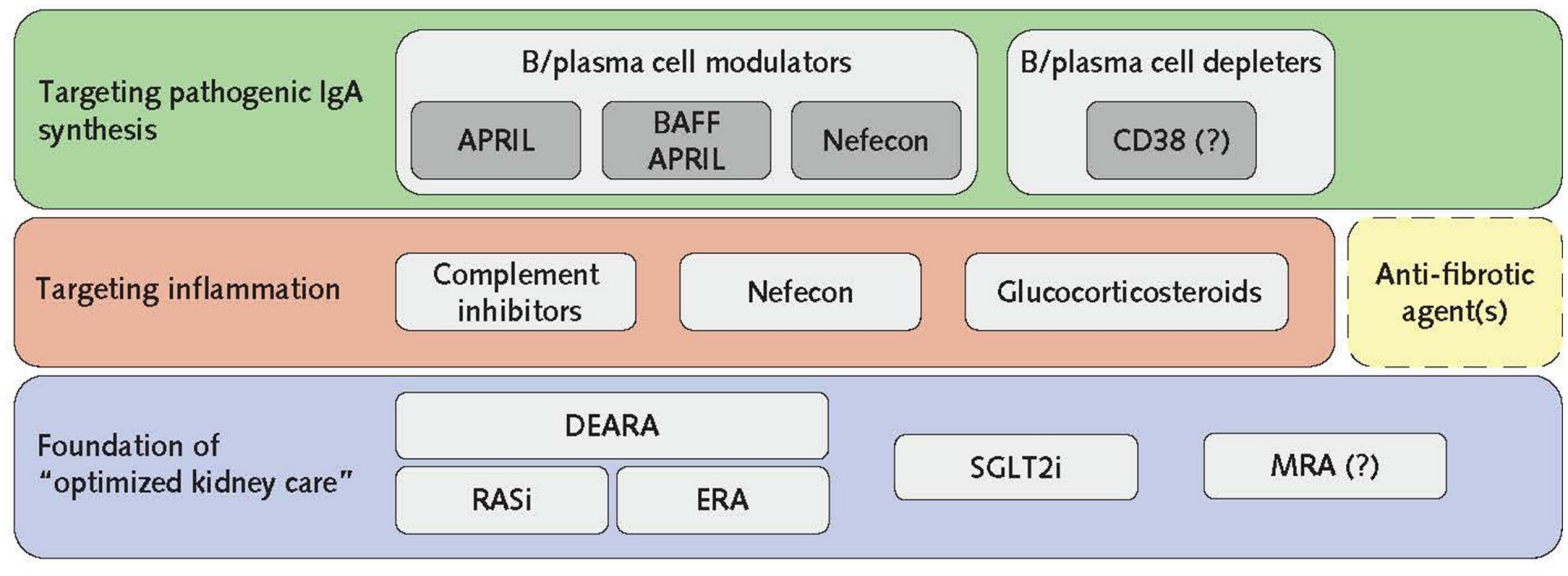
The pillars of IgAN treatment. To gain control of disease progression, the foundational basis of optimized kidney care should be initiated in combination with B/plasma cell-directed agents to switch off pathogenic IgA production and anti-inflammatory agents immediately after diagnosis. It should be noted that FIND-CKD investigating finerenone in IgAN is ongoing, and therefore MRA inclusion as a pillar of IgAN treatment remains to be confirmed. The inclusion of anti-fibrotic agents (dashed box) in this multilevel treatment strategy is aspirational as none are currently in clinical trials for IgAN. Once remission is achieved, therapies not required for maintenance may be removed with ongoing monitoring of eGFR and proteinuria. APRIL, a proliferation-inducing ligand; BAFF, B-cell activating factor; CD38, cluster of differentiation 38; DEARA, dual endothelin angiotensin receptor antagonist; eGFR, estimated glomerular filtration rate; ERA, endothelin receptor antagonist; IgAN, IgA nephropathy; MRA, mineralocorticoid receptor antagonist; RASi, renin–angiotensin system inhibitors; SGLT2i, sodium–glucose cotransporter-2 inhibitors.

With the goal to stop, or reduce, nephron loss immediately upon diagnosis, multitargeted treatment should commence as soon as possible. The current process, as with many causes of CKD, is the sequential addition of therapies following treatment failure and disease worsening, meaning nephron loss continues while additional therapies are slowly utilized. An alternative approach to maximize nephron preservation would be to simultaneously deal with the CKD and immunological drivers of nephron loss using treatment combinations (Figure 2), delivering a multitargeted approach to pathogenic IgA production, inflammation and the maladaptive responses that have become established in response to existing nephron loss.

As most patients with IgAN present with established CKD, optimized supportive kidney care is essential. This requires lifestyle modification advice alongside optimization of BP, CV risk reduction, and commencement of RASi, with the potential addition of an ERA, SGLT2i, and MRA. Alternatively, the combined antagonism of AT_1_R and ET_A_R, provided by the DEARA sparsentan, with addition of an SGLT2i and an MRA. Hypotension may be a limiting factor to starting all of these drugs in young IgAN patients and further study will be required to understand the best approach for such a strategy. Whether it is better to use a maximal dose of RASi and add an ERA (or switch to DEARA), SGLT2i, and/or MRA, depending on tolerability, or whether it is better to have the patient on low/medium doses of all drugs to gain from their different mechanisms of action, akin to the European Society of Cardiology’s 2021 Heart Failure guideline for initiation of the “Four Pillars” of treatment (69), needs to be established.

When addressing the immunological drivers for nephron loss, like all other forms of inflammatory immune-mediated glomerular disease, such as anti-neutrophil cytoplasmic antibody (ANCA)-associated vasculitis (AAV) and lupus nephritis, combined therapies to tackle inflammation and pathogenic antibody/autoantibody synthesis should be initiated at diagnosis (69). As with the treatment of AAV (70) the induction of remission in IgAN may require treatment combinations which are then stripped back following achievement of remission, as the maintenance phase begins. It is likely that once pathogenic IgA production is controlled there will be less reliance on anti-inflammatory drugs and complete withdrawal may be possible. Rather than aiming for complete withdrawal of B/plasma cell modulators/depleters, it may be necessary to consider cyclical treatments that would maximize efficacy while limiting adverse events and toxicity, as is currently employed in AAV with cyclical rituximab treatment to maintain long-term remission.

## Conclusion

The goal when treating patients with IgAN should be to save all remaining nephrons and prevent kidney failure in the patient’s lifetime if possible. This requires simultaneously addressing the maladaptive responses to nephron loss that are seen in all causes of CKD, and the specific immunological drivers of IgAN. With the great strides that have been made over the past 5 years, and ongoing clinical trials, the next few years should see a comprehensive IgAN treatment armamentarium become available. Determining how we use this wealth of treatment options will become increasingly important.
